# New Approach to the Old Challenge of Free Flap Monitoring—Hyperspectral Imaging Outperforms Clinical Assessment by Earlier Detection of Perfusion Failure

**DOI:** 10.3390/jpm11111101

**Published:** 2021-10-27

**Authors:** Daniel G. E. Thiem, Paul Römer, Sebastian Blatt, Bilal Al-Nawas, Peer W. Kämmerer

**Affiliations:** Department of Oral and Maxillofacial Surgery, University Medical Centre Mainz, 55131 Mainz, Germany; paul.roemer@unimedizin-mainz.de (P.R.); sebastian.blatt@unimedizin-mainz.de (S.B.); bilal.al-nawas@unimedizin-mainz.de (B.A.-N.); peer.kaemmerer@unimedizin-mainz.de (P.W.K.)

**Keywords:** HSI, objective, hyperspectral signature, timely recognition, reconstruction, head and neck, non-invasive, non-contact

## Abstract

In reconstructive surgery, free flap failure, especially in complex osteocutaneous reconstructions, represents a significant clinical burden. Therefore, the aim of the presented study was to assess hyperspectral imaging (HSI) for monitoring of free flaps compared to clinical monitoring. In a prospective, non-randomized clinical study, patients with free flap reconstruction of the oro-maxillofacial-complex were included. Monitoring was assessed clinically and by using hyperspectral imaging (TIVITA™ Tissue-System, DiaspectiveVision GmbH, Pepelow, Germany) to determine tissue-oxygen-saturation [StO_2_], near-infrared-perfusion-index [NPI], distribution of haemoglobin [THI] and water [TWI], and variance to an adjacent reference area (Δreference). A total of 54 primary and 11 secondary reconstructions were performed including fasciocutaneous and osteocutaneous flaps. Re-exploration was performed in 19 cases. A total of seven complete flap failures occurred, resulting in a 63% salvage rate. Mean time from flap inset to decision making for re-exploration based on clinical assessment was 23.1 ± 21.9 vs. 18.2 ± 19.4 h by the appearance of hyperspectral criteria indicating impaired perfusion (StO_2_ ≤ 32% OR StO_2_Δreference > −38% OR NPI ≤ 32.9 OR NPIΔreference ≥ −13.4%) resulting in a difference of 4.8 ± 5 h (*p* < 0.001). HSI seems able to detect perfusion compromise significantly earlier than clinical monitoring. These findings provide an interpretation aid for clinicians to simplify postoperative flap monitoring.

## 1. Introduction

In reconstructive oral and maxillofacial surgery, free flap transfer represents one of the most important and frequently performed methods for defect reconstruction of the head and neck region. Flap survival as the primary criterion for success after free flap transfer is generally considered to be very good at approximately 96% [1]. However, this is largely based on studies using less complex flap types such as fasciocutaneous radial or ulnar forearm flaps (R/UFFF) and does not generally apply to more compound flaps such as the osteocutaneous fibular flap (8% failure), scapular flap (6% failure), anterolateral thigh (ALT) or gracilis flap (5% failure) [2,3,4]. This is in contrast to the results of a recent study which showed flap survival of 98% in 157 fibular flaps used for mandibular reconstruction [5]. In addition to the flap type and its complexity, there are numerous other relevant factors (e.g., duration of surgery >8 h, need for intraoperative re-anastomosis, anatomically complex flap sites, challenging microanastomoses, arterial > venous thrombosis) that may contribute to the need for flap revision or even complete early flap failure [6]. Partial and complete flap loss, mainly due to impaired perfusion (venous > arterial), means a significant increase in morbidity and mortality for the affected patients due to prolonged wound healing, necessary second interventions, delay of adjuvant therapy (radio- and/or chemotherapy) and prolonged hospital stay [7,8]. In addition, the above-mentioned complications lead to a relevant additional financial burden on health care systems [9]. In this context, close perioperative flap monitoring has been established as the only effective tool allowing early detection of malperfusion and thus providing the possibility of timely re-exploration. Although several valid monitoring methods have been developed in recent years, clinical assessment, though subjective and poorly reproducible, is still considered the gold standard for flap monitoring [10]. The medical application of hyperspectral imaging (HSI) is an overall new and still quite unexplored field. Previous studies by our group have demonstrated the successful usage of medical hyperspectral imaging in the fields of wound diagnostics [11], perfusion monitoring after microsurgical anastomotic suturing in the rat hind limb and visualisation and quantification of the vasoactive effect of vasoconstrictor-containing local anaesthetics [12,13]. Following preliminary animal experiments [12], we were able to demonstrate the successful use of HSI to monitor free flaps in humans as part of a feasibility study [1]. The main limitation of this feasibility study was the limited number of compromised flaps, being the relevant variable to evaluate monitoring techniques. Therefore, the aim of this clinical study was to compare HSI and clinical monitoring in terms of their ability for early detection of impaired free flap perfusion.

## 2. Materials and Methods

### 2.1. Patients

In this prospective, non-randomized, clinical study, patients with free flaps for reconstruction of the oro-maxillofacial complex were included. The patient population consists of 40 males and 23 females. The average patient age was 62.2 ± 12.6 years. Due to its design, the sensor unit of the hyperspectral camera system was not able to image the posterior portion of the oropharynx. Therefore, free flaps of the anterior-lateral region of the maxilla and mandible, as well as exposed flaps of the facial region, were included in the present study. All flaps that did not have regular monitoring (t0–t10 for HSI and t4–t10 for clinical monitoring) were not included.

### 2.2. Monitoring

#### 2.2.1. Clinical Monitoring

**Figure 1:**
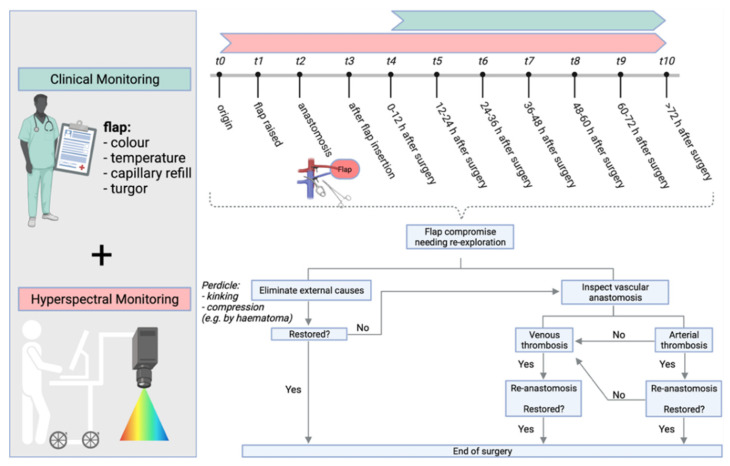
Graphical study protocol shows the measurement time points (t0–t3) and time intervals (t4–t10), as well as the process of decision making for re-exploration surgery. Created with BioRender.com (accessed on 13 October 2021).

Clinical monitoring started with the first postoperative control (t4) and was always performed prior to hyperspectral imaging to avoid examination bias. The measurement time points are shown in Figure 1. In each case, clinical parameters of flap perfusion (color, temperature, re-capillarization time and tissue turgor) were assessed and documented by an experienced physician. Point values were assigned to each clinical category (color, temperature, re-capillarization time, turgor). Criteria for surgical re-exploration were met if total was ≥9 OR two variables scored 6 points OR flap color remained pale white or blue >60 min AND re-capillarization time was not detectable or <1 s (Table 1). Because clinical monitoring is still considered the gold standard, the final decision for re-exploration was always based on clinical assessment.

#### 2.2.2. Hyperspectral Monitoring

**Figure 2:**
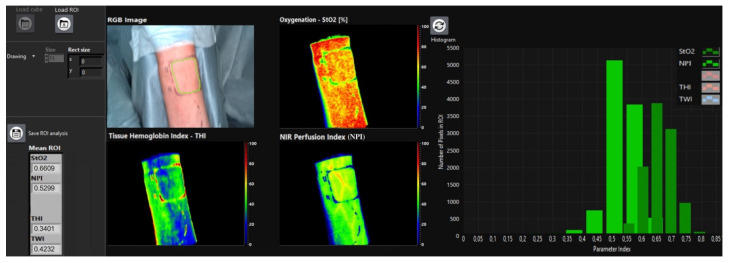
HSI shows the blood flow measured in the left ulnar flap raise site (t0) with the region of interest (ROI) marked manually (green line, RGB image). The quality of blood flow is indicated by false colors ranging from blue (low) to red (high). On the left, quantification of ROI is listed and shows mean values of the respective parameters (StO_2_, NPI, THI, TWI). The number of assessed pixels and the corresponding amount of StO_2_ and NPI within ROI (*Y*-axis) is represented as a bar chart.

In this study, a hyperspectral sensor system (TIVITA^TM^ Tissue System, Diaspective Vision GmbH, Pepelow, Germany) was used. The HSI sensors generate a three-dimensional (3D) data cube (hyperspectral cube), with the spatial information contained in the first two dimensions (resolution: 0.1 mm/pixel at 50 cm distance) and the spectral information in the third dimension (resolution: 5 nm). The method includes conventional as well as spectroscopic approaches to capture both spatial and spectral information of an image scene. While conventional RGB methods (red, green and blue) cover a limited wavelength spectrum, HSI is able to process electromagnetic wavelength spectra > 740 nm [14]. Briefly, HSI is based on the assessment of contiguous spectra (i.e., light of different wavelengths) individually re-emitted by molecules. These physicochemical raw data are then processed by computerized algorithms, specific for the respective molecule of interest (hyperspectral signatures), particularly haemoglobin, oxygenated haemoglobin and water [15,16]. Following HS-image recording over 10 s (s), additional 8 s are needed to compute a RGD (red, green and blue) truecolor image and additional four pseudo-color images, representing the parameters: tissue oxygen saturation [StO_2_ (0–100%)], near infrared perfusion index [NPI as arbitrary units (0–100)] as well as distribution of haemoglobin [THI as arbitrary units (0–100)] and water [TWI as arbitrary units (0–100)] (Figure 2) [1,17]. Haemoglobin and its differentiation between its oxygenated and deoxygenated form plays a central role in HSI perfusion monitoring [15]. Since the absorbance of haemoglobin in the range from 570 to 590 nm is high, electromagnetic radiation of a shorter wavelength shows a lower penetration depth into tissue, thus microcirculation is detected at a depth up to 1 mm. StO_2_ describes the relative oxygen saturation of blood in the microcirculatory system within superficial tissue layers, captures arterial and venous blood, and shows changes in oxygen supply and consumption directly in the tissue area measured. Thus, StO_2_ represents the tissue oxygen saturation, which is mainly based on the blood volume in the venous part (75%) of the microcirculation and its oxygen saturation after delivery of oxygen to the tissue. Reference values are between 50–70% [18]. However, there are no thresholds, although corresponding studies are currently being conducted. Near-Perfusion-Index (NPI) describes the quality of blood flow which is determined by the relative oxygen saturation of the haemoglobin and the relative haemoglobin content in deep tissue layers (4 to 6 mm) [1]. In the software from the manufacturer, parameters are displayed in false colors from red = high, through yellow and green, to blue = low. The Tissue-Haemoglobin-Index (THI) describes the relative amount of haemoglobin in the microcirculatory system. This parameter gives information on inflow and/or outflow disorders. Tissue Water Index (TWI) describes the relative water content in the assessed region of interest. We have described the importance of the parameters and their combination for perfusion assessment in detail in a previous publication [1].

### 2.3. Statistics

Raw data sets were saved in Excel^®^ sheets (Microsoft Corporation, Redmond, WA, USA) and subsequently transferred into SPSS Statistics^®^ (version 23.0.0.2, MacOS X; SPSS Inc., IBM Corporation, Armonk, NY, USA). Data were expressed as mean (m), standard deviation (SD±), minimum (min), maximum (max) and standard error of the mean (SEM). Normal distribution was checked using non-parametric Shapiro–Wilk-test(+) and Kolmogorov–Smirnov Test. In addition to the descriptive analysis, the dependency analysis included tests to detect/exclude differences and correlations. Results were analysed for statistical significance by the use of analysis of variance (ANOVA(#)), unpaired non-parametric Mann–Whitney U-tests($), Wilcoxon Signed Ranks test(§) and Students’ *t*-test(*). To investigate whether the means of several dependent samples differ, Wilcoxon matched-pairs signed rank tests(**) were performed. Correlations between two categorical variables were tested using the Pearson Chi-Square Test^(+)^ or, in the case of expected cell frequencies < 5, using Fisher’s Exact Test^(++)^. The eta-squared coefficient as a measure of correlation measures the extent to which the total variance of a dependent metric variable is explained by an independent nominal variable. The partial Eta square (Eta) shows how much % of the variation of “Duration of surgery” can be explained by the revision status (Group-1 vs. Group-2). The *p*-values of ≤0.05 were termed significant. Line charts with plotted means ±SD, pie charts, aligned dot plots and boxplots were used for illustration purposes. Due to the small number of similar studies, as well as the lack of cut-off values, case number planning could not be performed [19,20].

## 3. Results

A total of 54 primary and 11 secondary reconstructions were performed. Flaps included Radial-(RFF, 24) and Ulnar-(UFF, 16) Forearm flaps, Osteocutaneous Fibula flaps (OMFF, 16), Latissimus Dorsi flaps (LDF, 4), Osteocutaneous Scapula flaps (OMSF, 3) and 2 Upper Arm flaps (UAF). Affected regions included the tongue (4), cheek (18), floor of the mouth (10), alveolar ridge (2), soft (3) and hard palate (4), mandible (18), midface (4) and neurocranium (2). Recipient vessels were as follows: superior thyroid (43) (end-to-end), lingual (11) (end-to-end), external carotid (7) (end-to-side) and the facial artery (4) (end-to-end). Out of 65 flaps (two patients received 2 microvascular flaps each), re-exploration was performed in 19 cases (Group-2^(R+)^) due to a clinically apparent perfusion disturbance, of which in one case no cause could be found intraoperatively. In the latter, because there was improved flap perfusion after reopening of the neck in both, clinical assessment and HSI, this case was considered kinking of the pedicle. A total of seven complete flap failures occurred, resulting in a salvage rate of 63% regardless of cause (haematoma, kinking, arterial or venous thrombosis). There was no correlation between the reconstruction regime (primary or secondary)^(++)^, the irradiation status^(++)^, the arterial recipient vessel type^(+)^ or the duration of surgery (Eta = 0.06) and the occurrence of poor perfusion (need for revision) (Table 2).

### 3.1. Monitoring

#### 3.1.1. Clinical Monitoring Characteristics

**Figure 3:**
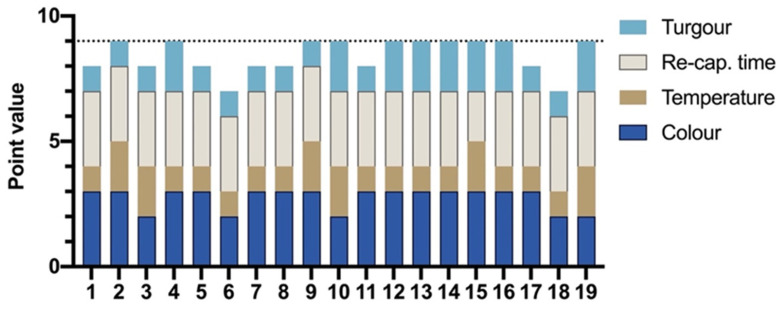
Bar chart shows the point total of clinical assessment from each impaired flap (Group-2(R+) that led to re-exploration.

The true-positive rate of clinical assessment for detection of perfusion defect was 100% with 19/19 flaps. The distribution of scores obtained using the clinical scoring system is shown in Figure 3.

#### 3.1.2. General HSI Characteristics for Non-Revised and Revised Flaps

##### Oxygen Saturation of Haemoglobin (StO_2_)

**Figure 4:**
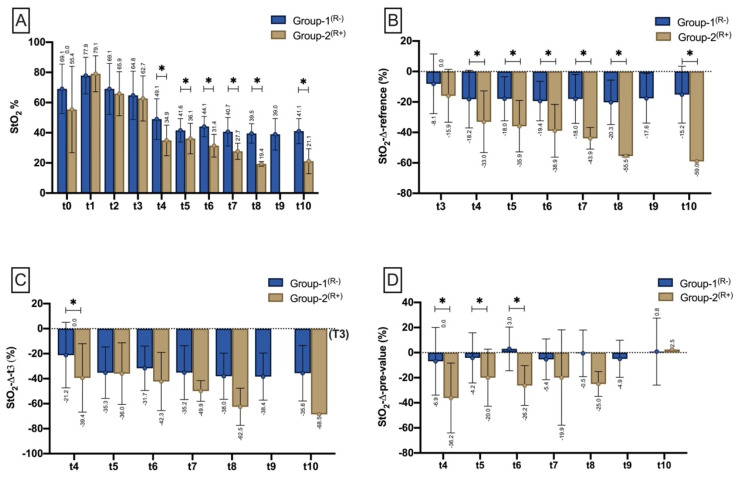
Bar chart with means (±SD) show StO_2_ at different measurement timepoints (T0–T3)/time intervals (t4–t10) as (**A**) (StO_2_ mean), (**B**) StO_2_ drop to reference (%) StO_2_ drop to the respective reference site (StO_2_Δreference), (**C**) StO_2_ drop to t3 (StO_2_Δt3), and (**D**) in relation to the pre-measurement (StO_2_Δpre-value). Means are shown scalar. Asterisk (*) marks existing significance.

Except t0 (origin), t1 (flap raise), t2 (anastomosis) and t3 (flap inset), StO_2_ was significantly lower in Group-2^(R+)^ at any time after flap inset including t4 (0–12 h; *p* < 0.001), t5 (12–24 h; *p* = 0.034), t6 (24–36 h; *p* < 0.001), t7 (36–48 h; *p* = 0.008), t8 (48–60 h, *p* < 0.001), and t10 (>72 h, *p* = 0.004) (Figure 4A). Compared with the reference site (StO_2_Δreference), StO_2_ decreased (%) significantly in Group-2^(R+)^ during t4 (*p* = 0.004), t5 (*p* < 0.001), t6 (*p* < 0.001), t7 (*p* = 0.002), t8 (*p* = 0.002), and t10 (*p* = 0.006^(^*^)^) compared to Group-1^(R−)^ (Figure 4B). Compared with flap inset (t3), StO_2_ significantly (*p* = 0.011^(^*^)^) decreased at t4 in Group-2^(R+)^ when compared to Group-1^(R−)^ (Figure 4C). Regarding the difference to the pre-value (StO_2_Δpre-value), StO_2_ decreased significantly in Group-2^(R+)^ during t4 (*p* < 0.001^(^*^)^), t5 (*p* = 0.021^(^*^)^), and t6 (*p* < 0.001^(^*^)^) when compared to Group-1^(R−)^, respectively (Figure 4D).

##### NIR-Perfusion Index (NPI)

**Figure 5:**
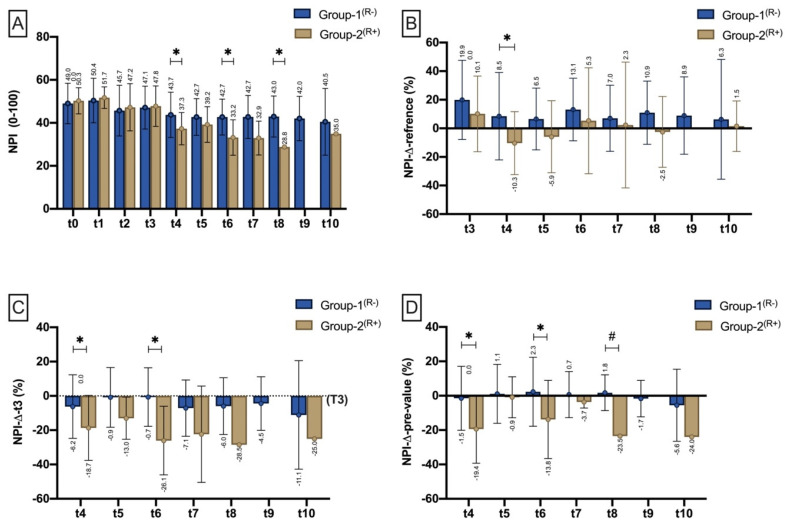
Bar chart with means (±SD) show NPI at different measurement timepoints (t0–t3)/time intervals (t4–t10) as (**A**) (NPI mean), (**B**) NPI drop to reference (%) NPI drop to the respective reference site (NPIΔreference), (**C**) NPI drop to t3 (NPIΔt3), and (**D**) in relation to the pre-measurement (NPIΔpre-value). Means are shown scalar. Asterisk (*) marks existing significance. ANOVA (#).

There was no difference in Near-Infrared Perfusion Index (NIR-P) between Group-1^(R−)^ and Group-2^(R+)^ before flap harvesting (T0), after flap raise (t1), after microvascular anastomosis (t2), and after flap insertion (t3). Within the first 12 h after flap inset, the Near-Infrared Perfusion-Index (NPI) was significantly lower in Group-2^(R+)^ compared to Group-1^(R−)^ (*p* = 0.024^(^*^)^). The same was seen within t6 (*p* = 0.008^(^*^)^) and t8 (*p* = 0.045^(^*^)^), with the latter period containing only two compromised flaps that were subsequently re-explored (Figure 5A). Compared with the reference measurement site, Group-2^(R+)^ revealed a significantly greater decrease in NPI (%) (NPIΔreference) at t4 (*p* = 0.023^(^*^)^) compared to Group-1^(R−)^ (Figure 5B). Compared with t3 (NPIΔt3), NPI decreased significantly more in Group-2^(R+)^ at t4 (*p* = 0.016^(^*^)^) and t6 (*p* = 0.011^(^*^)^) than in Group-1^(R−)^ (Figure 5C). Compared with the pre-measurement (NPIΔpre-value), the NPI drop was significantly higher in Group-2^(R+)^ within the measurement intervals t4 (*p* < 0.001^(^*^)^), t6 (*p* = 0.044^($)^) and t8 (*p* = 0.002^(^*^)^) than in Group-1^(R−)^ (Figure 5D).

##### Tissue-Haemoglobin-Index (THI)

**Figure 6:**
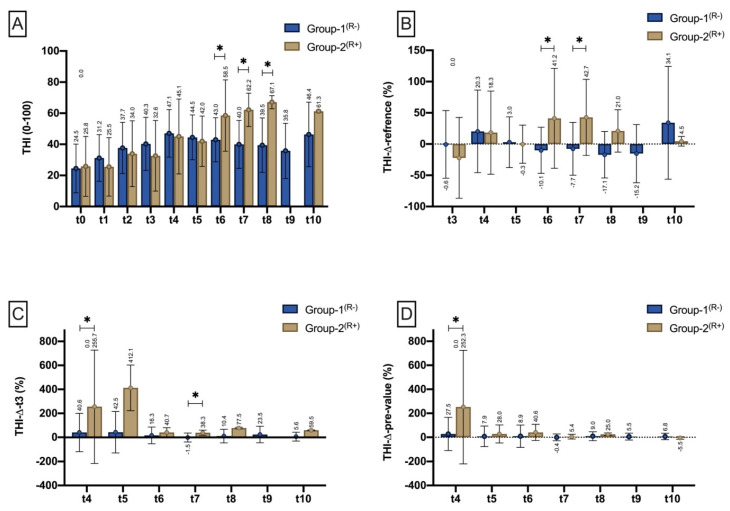
Bar chart with means (±SD) show THI at different measurement timepoints (t0–t3)/time intervals (t4–t10) as (**A**) (THI mean), (**B**) THI drop to reference (%) THI drop to the respective reference site (THIΔreference), (**C**) THI drop to t3 (THIΔt3), and (**D**) in relation to the pre-measurement (THIΔpre-value). Means are shown scalar. Asterisk (*) marks existing significance.

At the measurement time points (T0-t3)/within the measurement intervals (t4 and t5), the Tissue-Haemoglobin Index (THI) was not different between Group-2^(R+)^ and Group-1^(R−)^. At t6 (*p* = 0.010^(^*^)^), t7 (*p* = 0.006^(^*^)^) and t8 (*p* = 0.033^(^*^)^), THI was significantly increased in Group-2^(R+)^ (Figure 6A). In proportion to the reference site (THIΔreference), THI was significantly increased in Group-2^(R+)^ compared with Group-1^(R)^ during t6 (*p* = 0.048^($)^) and t7 (*p* = 0.048^($)^) (Figure 6B). Compared with flap inset (THIΔt3), THI was significantly higher in Group-2^(R+)^ than in Group-1^(R−)^ during t4 (*p* = 0.011^($)^) and t7 (*p* = 0.012^($)^) (Figure 6C). Compared with the pre-measurement (THIΔpre-value), the percentage increase in THI was significantly higher in Group-2^(R+)^ within the measurement interval t4 (*p* < 0.001^($)^) than in Group-1^(R−)^ (Figure 6D).

##### Tissue-Water-Index (TWI)

**Figure 7:**
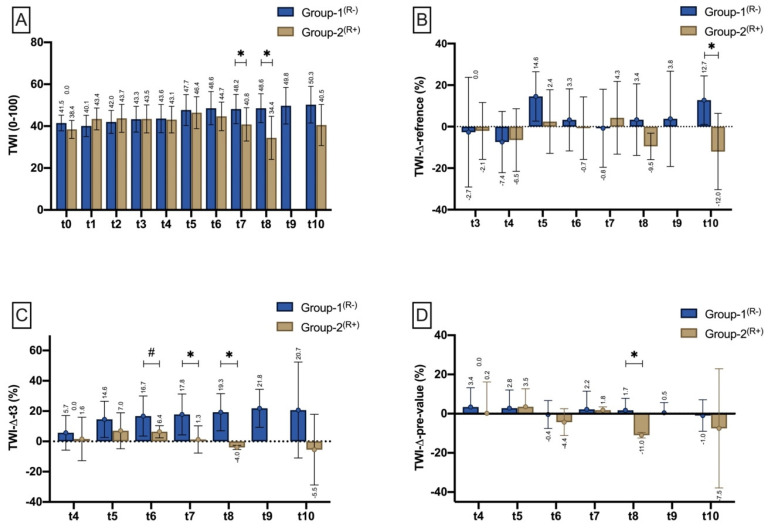
Bar chart with means (±SD) show TWI at different measurement timepoints (t0–t3)/time intervals (t4–t10) as (**A**) (TWI mean), (**B**) TWI drop to reference (%) TWI drop to the respective reference site (TWIΔreference), (**C**) TWI drop to t3 (TWIΔt3), and (**D**) in relation to the pre-measurement (TWIΔpre-value). Means are shown scalar. Asterisk (*) marks existing significance. ANOVA (#).

From t0 to t6, there was no difference in the water content (TWI) comparing Group-1^(R−)^ and Group-2^(R+)^. At t7 and t8, TWI was found to be significantly lower (*p* = 0.045^(^*^)^; *p* = 0.007^(^*^)^) in the group of poorly perfused flaps (Figure 7A). Compared with the reference site (TWIΔreference), TWI was significantly lower at t8 (*p* = 0.035^($)^) in Group-2^(R+)^ than in Group-1^(R−)^ (Figure 7B). Compared to flap inset (THIΔt3), TWI was significantly lower in Group-2^(R+)^ at t6 (*p* = 0.015^($)^), t7 (*p* = 0.008^($)^) and t8 (*p* = 0.002^($)^) (Figure 7C). Compared with the pre-measurement (TWIΔpre-value), TWI decreased significantly more in Group-2^(R+)^ at t8 (*p* = 0.002^($)^) (Figure 7D).

##### Duration until Signs of Malperfusion

**Figure 8:**
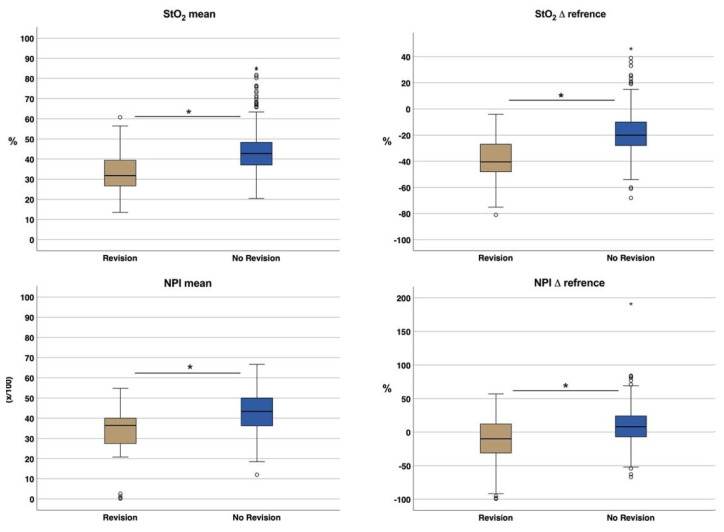
Boxplot showing the distribution of StO2, StO_2_Δreference, NPI and NPIΔreference comparing flaps with and without revision following poor perfusion. Asterisk (*) marks existing significance.

Independent of the time point/interval, mean values of StO_2_ and NPI as well as their drop rate (Δreference) differed significantly between Group-1^(R-)^ and Group-2^(R+)^ (Figure 8). StO_2_, as well as StO_2_Δreference were significantly lower in Group-2^(R+)^ (32.6% ± 9.8; −38.1% ± 18.2) than in Group-1^(R−)^ (43.2% ± 10.3; −18.3% ± 15.9) (*p* < 0.001). The same was seen with NPI (Group-1^(R−)^: 42.8 ± 9.8; Group-2^(R+)^: 32.9 ± 12.8) and NPIΔreference (Group-1^(R−)^: 8.8 ± 25.7; Group-2^(R+)^: −13.4% ± 36.9) (*p* < 0.001^(^*^)^).

**Figure 9:**
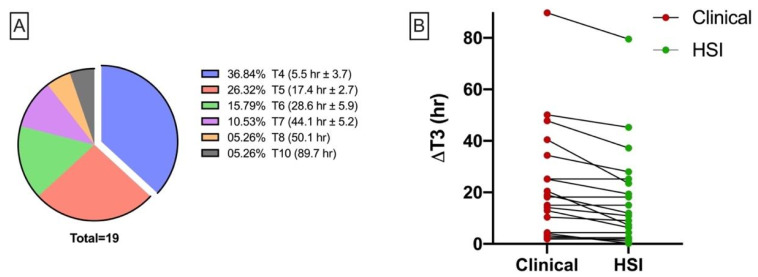
(**A**) Pie chart showing actual duration to clinical manifestation of flap malperfusion and subsequent re-exploration with associated time interval. (**B**) Aligned dot plot shows duration (h) from flap insertion to clinical (left) and hyperspectral detection of flap malperfusion.

Of a total of 19 compromised flaps, 12 (36.8%) occurred within the first 24 h postoperatively. The exact distribution is shown in Figure 9A. To calculate the duration from flap inset (t3) to the detection of malperfusion in HSI, the mean values of StO_2_ (32.6%), StO_2_Δreference (−38.1%), NPI (32.9) and NPIΔreference (−13.4%) were used for dichotomization. If there was a negative deviation (<StO_2_; <NPI; >StO_2_Δreference; >NPIΔreference) from one of the parameters, the respective measurement time was counted as the detection time. Overall, the mean time from flap inset to decision making for re-exploration based on clinical assessment was 23.1 ± 21.9 h. In contrast, the average time from flap insertion to the appearance of hyperspectral criteria of inferior perfusion (StO_2_ ≤ 32% OR StO_2_ diff > −38% OR NPI ≤ 32.9 OR NPI diff. ≥ −13.4%) was 18.2 ± 19.4 h, resulting in a difference of 4.8 ± 5 h (*p* < 0.001^(^**^)^) (Figure 9B).

## 4. Discussion

In this study, monitoring of free flap perfusion in the head and neck region was compared between clinical assessment and hyperspectral imaging. As the major finding, malperfusion could be detected at a mean of 4.8 h earlier with the help of Hyperspectral Imaging (HSI) when compared to clinical examination. In addition, general information on the perfusion characteristics of the included flap types were presented.

Postoperative flap monitoring is a key component for successful free tissue transfer whereby early detection of malperfusion is the pivotal criterion for treatment success as only early detection can ensure timely re-exploration to avoid flap failure [21]. One measure of this is the overall salvage rate which is reported to be from 60% up to 80% on average (63% in the present study) [22,23]. However, this neglects the subdivision according to the underlying cause (venous or arterial), with the salvage rate being significantly lower for arterial thrombosis [24]. To overcome the issue of delayed re-exploration due to late detection of malperfusion, several monitoring methods have been developed during the last decades, including the implantable Doppler, color duplex sonography, near-infrared spectroscopy, laser Doppler flowmetry, fluorescence angiography and microdialysis [10,25,26,27,28]. The ideal flap monitoring technique should be continuous, accurate, cost-efficient, non-invasive, safe, objective, recordable, reliable, reproducible, sensitive, highly spatially resolved, easy to use/interpret and applicable to all flap types [29]. As currently no single traditional monitoring technique meets all these requirements, clinical examination, as the least reproducible and little to no objective technique, still remains the most frequently used [10]. However, clinical assessment depends on evaluator experience and it is only reliable when the flap color changes significantly into pale or blueish [30]. In addition, technical monitoring support is only used by 30% of the surgeons whereas the Doppler (handheld or implanted) is the most commonly used method for free flap monitoring [31]. In addition to increased costs due to consumables and acquisition, the experience-dependent classification of measurement results is also cited as a limitation of technology-based monitoring techniques [30]. When color duplex ultrasound is used, microanastomotic vessels can also be visualized in embedded flaps. It is a non-invasive and quantitative flap monitoring technique, whereby its use requires special training. The implantable doppler is placed distal to an anastomosis in contact to the vessel, to allow continuous measurement of blood flow [32]. However, it is an invasive technique that does not allow quantitative measurement. Laser Doppler flowmetry (LDF) is also frequently used for monitoring of free flaps whereby probes are implanted or applied to the flap surface. In this context, Yoshino et al. could not distinguish between arterial and venous malperfusion when monitoring 37 intraoral free flaps with LDF [33]. In contrast, Muecke et al. demonstrated in an animal study that the combination of at least two different technical monitoring methods (ICG and flowmeter) improved the monitoring of critical and/or buried flaps, whereas the use of the multispectral technique (O2C) diminished the predictive value [34]. In contrast, Hölzle et al. described the successful use of O2C (Oxygen-to-see, LEA-Medizintechnik GmbH, Giessen, Germany), a device combination of laser doppler flowmeter and tissue spectrophotometry, to monitor free flaps and to detect flap malperfusion at an early stage [35]. Compared to multispectral methods, the use of the complete and high-resolution spectrum (hyperspectral) results in significantly increased reliability and reproducibility of parameter determination [36]. Providing non-contact, non-invasive measurements, HSI allows perfusion monitoring status in different tissue layers/depths through pictorial representation of parameters calculated from the spectra (tissue oxygenation saturation (StO_2_), Near-Infrared Perfusion Index (NPI), Tissue Haemoglobin Index (THI), and Tissue Water Index (TWI)). Using the THI, additional conclusions can be drawn about the underlying cause of perfusion failure (venous versus arterial perfusion compromise) [1]. However, in the present study there was no significant correlation between StO_2_ or NPI and THI. There were no signs of impaired flap perfusion at flap origin (t0), at flap/pedicle preparation (t1), directly after anastomosis (t2) or after flap inset (t3), as StO_2_, NPI, THI and TWI did not differ significantly between Group 1^(R−)^ and 2^(R+)^. The characteristics of the blood flow dynamic of free flaps prior to the actual tissue transfer as well as its possible reasons have already been presented in a previous publication [1]. During t4, StO_2_ was significantly lower on average in Group 2^(R+)^ (34.9 ± 10%) than in Group 1^(R−)^ (49.1 ±13.5% *p* < 0.001). This, in turn, is in line with the earlier findings of our group, as well as with those of Kohler et al., defining 40% as the lower limit of StO_2_ in normal perfused flaps [1,37]. In both of these pilot studies, the low number of malperfused flaps (<10) must be taken into account with regard to their significance. Deep tissue perfusion (represented by NPI) was also significantly lower (37.3 ± 7.5 *p* < 0.001) in Group 2^(R+)^ within the first 12 h (t4) in contrast to Group 1^(R−)^ (43.75 ± 10.52). No significant differences were found for THI and TWI.

Since StO_2_ and NPI are not independent of systemic total haemoglobin (regarding the relationship between Hb and StO_2_), we consider the drop rate (Δreference), as a measure of the systemic blood flow situation (adjacent reference site), providing high predictive value. This is in accordance with Keller et al. who stated the drop rate as a meaningful instrument [38]. In comparison between group 1^(R−)^ and 2^(R+)^, both StO_2_^Δ^^reference^ and NPI^Δ^^reference^ were overall (t4–t10) significantly lower in group 2^(R+)^ (Figure 8). Previous studies on medical HSI were able to investigate its application in the field of visceral surgery, as well as plastic reconstructive surgery. In this context Barberio et al. implemented HSI as an intraoperative surgical guidance tool, using its capability of accurate detection and visualization of perfusion changes in the region of ischemic bowel segments [39]. The same group was able to demonstrate the successful usage of HSI and confocal laser endomicroscopy (CLE) for perfusion monitoring in esophageal surgery [40]. In a preclinical animal study, Chin et al. demonstrated the successful use of hyperspectral imaging for early detection of malperfusion in random axial flaps [41], as well as Grambow et al. revealed real-time perfusion monitoring of the rats’ hind limb after vessel transection and re-anastomosis [12]. Recent approaches have been able to successfully perform automated tissue classification and differentiation ex- and in-vivo based on hyperspectral cubes using deep learning algorithms (neural networks and computer vision) [14,42,43].

Disadvantages of HSI are a relevant dependence on ambient illumination, as well as the lack of applicability in heavily pigmented individuals due to extended light absorption. The issue of illumination particularly affects intraoral skin islands, but these can probably be better examined in the near future with a newly developed endoscope variant of the system used. Furthermore, the number of microvascular flaps included must be mentioned as a study-specific limitation, although the crucial number of poorly perfused flaps (19), presents a valuable and representative collective of the main subject. While the sensitivity to detect malperfusion is the same for clinical monitoring and HSI (100%), we demonstrated that HSI indicates poor perfusion significantly earlier (4.8 h).

## 5. Conclusions

On average, evidence of critical flap perfusion occurred 4.8 h earlier in hyperspectral imaging when compared to clinical assessment. Therefore, our findings provide an interpretation aid for clinicians to simplify postoperative flap monitoring.

## Figures and Tables

**Table 1:** Clinical flap evaluation system with point values.

Flap Color	Flap Temperature	Re-Capillarization Time	Flap Turgor	Total
Pale white (3)	Cold (2)	Approx. 1 s (2)	Soft (2)	
Pink (1)	Body temperature (1)	>2 s (1)	Elastic (1)	
Red (2)	Superheated (2)	<1 s (3)	Plump (2)	
Blue (3)		No capillary refill (3)		

**Table 2:** Baseline data showing patient and flap characteristics.

	Group-1^(R+)^ No Revision	Group-2^(R+)^ Revision	Total (N)	*p*-Value
N	46	19	65	
Age	64.2 ± 11.7	53.6 ± 18		0.48^(^^+)^
Gender				0.10^(++)^
male	26 (63%)	15 (37%)	41	
female	20 (83%)	4 (17%)	24	
Indication				
Malignant	43 (73%)	16 (27%)	59	
Benign	1 (50%)	1 (50%)	2	
Chronic wound	2 (50%)	2 (50%)	4	
Flap types				0.27^(+)^
RFF	16 (66.6%)	8 (33.3%)	24	
UFF	13 (81%)	3 (19%)	16	
OMFF	13 (81.3%)	3 (18.7%)	16	
LDF	1 (25%)	3 (75%)	4	
OMSF	2 (66.6%)	1 (33.3%)	3	
UAF	1 (50%)	1 (50%)	2	
Reconstruction regime				0.72^(++)^
Primary reconstruction	39 (72%)	15 (28%)	54	
Secondary reconstruction	7 (63.6%)	4 (36.4%)	11	
After radiotherapy	6 (66.7%)	3 (33.3%)	9	0.71^(++)^
Recipient vessel (artery)				0.99^(+)^
Superior thyroid	30 (70%)	13 (30%)	43	
Lingual	8 (72.7%)	3 (27.3%)	11	
External carotid	5 (71%)	2 (29%)	7	
Facial	3 (75%)	1 (25%)	4	
Duration of surgery (minutes)	543.7 ± 126.5	527.3 ± 128.1		0.06^(Eta)^
Cause for malperfusion				
Venous thrombosis		8 (42.1%)	8	
Arterial thrombosis		8 (36.8%)	8	
Haematoma		3 (4.6%)	3	
Kinking		1 (5.3%)	1	

^+^ = Chi-Square test; ^++^ = Fisher’s Exact Test; ^Eta^ = Partial Eta square (explained in Section 2.3. *Statistics*).

## Data Availability

All raw data on which this study is based will be made available by the corresponding author upon request.
